# COX-2 modulates mammary tumor progression in response to collagen density

**DOI:** 10.1186/s13058-016-0695-3

**Published:** 2016-03-22

**Authors:** Karla Esbona, David Inman, Sandeep Saha, Justin Jeffery, Pepper Schedin, Lee Wilke, Patricia Keely

**Affiliations:** Department of Cell and Regenerative Biology, University of Wisconsin-Madison, Madison, WI USA; Institute for Clinical and Translational Research (ICTR), University of Wisconsin-Madison, Madison, WI USA; Department of Biostatistics and Medical Informatics, University of Wisconsin-Madison, Madison, WI USA; School of Medicine and Public Health, University of Wisconsin-Madison, Madison, WI USA; Department of Cell and Developmental Biology, School of Medicine, Knight Cancer Institute, Oregon Health and Science University, Portland, OR USA

## Abstract

**Background:**

High breast density is linked to an increased risk of breast cancer, and correlates with changes in collagen. In a mouse model of mammary carcinoma in the context of increased collagen deposition, the MMTV-PyMT/Col1a1^*tm1jae*^, there is accelerated mammary tumor formation and progression. Previous gene expression analysis suggests that increased collagen density elevates expression of PTGS2 (prostaglandin-endoperoxide synthase 2), the gene for cyclooxygenase-2 (COX-2).

**Methods:**

To understand the role of COX-2 in tumor progression within a collagen-dense microenvironment, we treated MMTV-PyMT or MMTV-PyMT/Col1a1^*tm1jae*^ tumors prior to and after tumor formation. Animals received treatment with celecoxib, a specific COX-2 inhibitor, or placebo. Mammary tumors were examined for COX-2, inflammatory and stromal cell components, and collagen deposition through immunohistochemical analysis, immunofluorescence, multiplex cytokine ELISA and tissue imaging techniques.

**Results:**

PyMT/Col1a1^*tm1jae*^ tumors were larger, more proliferative, and expressed higher levels of COX-2 and PGE2 than PyMT tumors in wild type (WT) mice. Treatment with celecoxib significantly decreased the induced tumor size and metastasis of the PyMT/Col1a1 tumors, such that their size was not different from the smaller PyMT tumors. Celecoxib had minimal effect on the PyMT tumors. Celecoxib decreased expression levels of COX-2, PGE2, and Ki-67. Several cytokines were over-expressed in PyMT/Col1a1 compared to PyMT, and celecoxib treatment prevented their over-expression. Furthermore, macrophage and neutrophil recruitment were enhanced in PyMT/Col1a1 tumors, and this effect was inhibited by celecoxib. Notably, COX-2 inhibition reduced overall collagen deposition. Finally, when celecoxib was used prior to tumor formation, PyMT/Col1a1 tumors were fewer and smaller than in untreated animals.

**Conclusion:**

These findings suggest that COX-2 has a direct role in modulating tumor progression in tumors arising within collagen-dense microenvironments, and suggest that COX-2 may be an effective therapeutic target for women with dense breast tissue and early-stage breast cancer.

**Electronic supplementary material:**

The online version of this article (doi:10.1186/s13058-016-0695-3) contains supplementary material, which is available to authorized users.

## Background

Breast cancer is the most common invasive cancer in women with upwards of 40,000 deaths annually in the USA [1]. Women who have over 75 % mammographic breast density have a more than four-fold increased risk for developing breast cancer, making it one of the most significant risk factors for this disease [2–6]. High breast density correlates with larger amounts of collagen fibers in the breast tissue [7], and a key feature of this density/collagen association is the presence of excessive and altered collagen structure and distribution. Our group has defined changes in collagen structure that manifest as bundles of straightened and aligned collagen fibers oriented perpendicular to a tumor boundary, termed tumor associated collagen signature-3 (TACS-3) [8], which are associated with decreased survival among patients with breast cancer [9]. In a transgenic mouse model with increased collagen deposition (mouse mammary tumor virus-polyomavirus middle T/carrying the collagenase transgene (MMTV-PyMT/Col1a1^*tm1jae*^)) the number and size of tumors and metastases increases three-fold [8, 10]. Despite the accumulation of data suggesting a role for increased collagen in mammary tumor progression, the molecular mechanisms for the increased risk and subsequent cancer development are unknown.

We previously found that culture of mammary epithelial cells in a high-density collagen matrix significantly upregulates *prostaglandin-endoperoxide synthase 2* (*PTGS2*), the gene for cyclooxygenase-2 (COX-2) [11, 12]. COX-2 over-expression is observed in 40 % of patients with invasive breast carcinoma and correlates with poor prognosis [13–15]. Unlike the constitutive activity of COX-1, COX-2 is an inducible enzyme that synthesizes prostaglandins, including prostaglandin E2 (PGE2), and is activated at sites of injury as part of the inflammation response [16]. Mammographically dense breast tissue has elevated COX-2 expression compared to breast tissue from women with low mammographic density [17]. In rodent models of mammary neoplasms, COX-2 over-expression promotes tumor formation and progression to metastasis, in addition to increased angiogenesis, cell migration, and invasion [12, 18–21]. High COX-2 expression correlates with increased levels of aligned collagen and both are the driving force for the development of ductal carcinoma *in situ* in a postpartum mammary gland involution mouse model. Moreover, treatment with non-steroidal anti-inflammatory drugs (NSAIDs), which inhibit cyclooxygenases, reverses this effect [12]. Celecoxib is a selective NSAID that specifically inhibits COX-2 and is the only COX-2 inhibitor currently approved by the Food and Drug Administration (FDA) for use in the USA [22]. Several studies have demonstrated that NSAIDs decrease the risk of cancer development [23–30]. Specifically, celecoxib prevents sporadic colorectal adenoma [31] and several clinical trials have evaluated the use of celecoxib alone or in combination with chemotherapy regimens in breast cancer settings [13]. Despite these associations, the role of COX-2 in collagen remodeling and in development of invasive breast cancer is still unclear.

In this report we tested the hypothesis that breast density promotes high COX-2 levels, which support tumor growth and progression. The goal of this study was to describe the role of COX-2, inflammation, and density in the breast tumor microenvironment. We found that COX-2 and PGE2 levels are elevated in the collagen dense (PyMT/Col1a1) tumors, and COX-2 inhibition with celecoxib decreases these expression levels. Treatment with celecoxib significantly diminished tumor growth and proliferation in the collagen dense tumors. Many cytokines were over-expressed in PyMT/Col1a1 tumors, and COX-2 inhibition reversed their over-expression. Results from this cytokine panel led us to look closer at different immune and stromal cell populations and their response to COX-2 inhibition in PyMT/Col1a1 and wild-type (WT) tumor microenvironments. We found that macrophage and neutrophil recruitment are enhanced in PyMT/Col1a1 tumors and enhancement was blocked by COX-2 inhibition. In addition, celecoxib decreased α-SMA^+^ fibroblast numbers in PyMT/Col1a1 tumors. Collagen deposition in both PyMT and PyMT/Col1a1 tumor microenvironments was diminished with celecoxib; however, normal mammary glands were not affected by COX-2 inhibition. Together, these findings suggest that COX-2 has a direct role in modulating tumor progression in dense matrices, which promote a more invasive cancer effect. COX-2 may be an effective therapeutic target for women with dense breast-tissue-associated breast cancer.

## Methods

### Mice and trial design

Mice were maintained and bred at the University of Wisconsin under the oversight of and with the ethical approval of the University of Wisconsin Animal Use and Care Committee (approved protocol # M01688). A therapeutic mouse model was used to evaluate the effects of high COX-2 expression in an advanced stage of mammary cancer (Fig. 2a). Nulliparous female MMTV-PyMT/Col1a1^*tm1jae*^, their PyMT counterparts bearing mammary tumors, Col1a1^*tm1jae*^ (no tumor), and their WT littermates were randomly assigned to a daily treatment of 0.2 mg (linear scale from 600 mg human dose or 10 mg/kg of body weight) celecoxib (Pfeizer Inc.) suspended in 5 % methyl cellulose or 5 % methyl cellulose alone (vehicle) at 11 weeks of age for a duration of 21 days. Dosage calculations were made for a 20-g mouse. Tissues were collected for study at 14 weeks of age.

To evaluate the effects of COX-2 inhibition with celecoxib in response to collagen density as a preventive breast cancer therapy, a preventive mouse mammary model was used (Fig. 8a). Female MMTV-PyMT/Col1a1^*tm1jae*^, their WT counterparts bearing mammary tumors, Col1a1^*tm1jae*^ (no tumor), and their WT littermates at 10 days of age were randomly assigned to treatment with celecoxib suspension or vehicle. Ten days of age is as early as neonate mice can be handled for oral administration of their assigned treatment, and is a developmental stage that precedes tumor formation in this model. First, neonate mice were orally fed with celecoxib 3.3 mg/kg of body weight (linear scale from 200 mg human dose) or vehicle every other day until they were weaned at 3 weeks of age. Dosage calculations were made for a 10-g mouse. At this low dose, celecoxib is not thought to interfere with development or cause other physiological complications in the pediatric population [32, 33]. At weaning, the dose was increased to 6.7 mg/kg of body weight (linear scale from 400 mg human dose) every other day until mice reached 9 weeks of age. Dosage calculations were made for a 20-g mouse. At 9 weeks of age, tumors were clearly palpable, and animals were killed for tissue analysis.

### Antibodies

The following antibodies were used for immunohistochemical analysis (IHC) and/or immunofluorescence (IF): COX-2 (Cayman 160126), PGE2 (Abcam ab2318), Ki-67 (Abcam ab15580), β-NGF (Abcam ab6199), IL-17A-R (LSBio LS-B6706), F4/80 (AbD Serotec MCA497R), Ly6g (Biolegend 127601), vimentin (Abcam ab92547) and alpha-smooth muscle actin (α-SMA) (Abcam ab5694).

### Histology, immunohistochemistry and immunofluorescence

For histology the tissues were fixed in 10 % formalin for 48 h followed by paraffin-embedding (formalin-fixed paraffin-embedded (FFPE)). Tissue sections were stained with hematoxylin and eosin (H&E). For IHC, FFPE tissues were deparaffinized as per standard, followed by dehydration and antigen retrieval with Citra Plus (Biogenex HK080-5 K) for 15 minutes, blocking with BLOXALL, avidin/biotin (Vector SP-6000 and SP-2001, respectively), and normal serum. Primary antibodies were incubated either overnight at 4 °C (anti-COX-2 or anti-PGE2 1:500) or were incubated for 1 h at room temperature (anti-Ki-67 1:200). Tissue sections were incubated with biotinylated rabbit IgG (Vector, BA-1100) for 10 minutes after 30-minute incubation with R.T.U. Vectastain kit Elite ABC (Vector PK-7100). For IF, tissue sections were treated as described above for 20 minutes to retrieve antigens and then were subjected to the TSA Plus kit for tissue labeling following the manufacturers’ protocols (Perkin Elmer, fluorescein NEL741E001KT, Cy 3.5 NEL744E001KT and Cy 5 NEL745E001KT). Briefly, primary antibodies were incubated as follows: COX-2 (1:1000, overnight); PGE2 (1:5000, 1 h); β-nerve growth factor (NGF) (1:6000, O/N); IL17RA (1:10000, 1 h); F4/80 (1:1000, 1 h); Ly6g (1:1000, 1 h); vimentin (1:1000, 1 h); α-SMA (1:1000, 1 h). Horseradish peroxidase (HRP)-conjugated anti-rabbit (Abcam, ab7090) or anti-rat (Abcam, ab7097) was added for 10 minutes after 10 minutes incubation with TSA Plus kit working solution including the desired fluorophore. Tissues underwent the antigen retrieval step for 20 minutes if the same tissue would be subjected to multiple labeling, before counterstaining with 4',6-diamidino-2-phenylindole (DAPI) for 2 minutes at 1:10000 (Life Technologies, D21490).

### Nuance and InForm software

IF and IHC image experiments were acquired using a Nuance microscope with × 20 objective and software version 3.0.12 (Perkin Elmer) with analysis as previously described [34]. Briefly, a spectral library was created using image cubes to define distinctive spectral curves for each fluorophore, chromogen, and counterstain to adjust for background effects and accurately quantify positive staining of biomarkers using InForm version 1.4.0 software (Perkin Elmer). This software analysis allows objective counting of cell populations and biomarkers and increases the accuracy of the statistical analysis. Algorithms for tissue and subcellular compartment separation were created by machine learning and all algorithms had precision above 95 % (Additional file 1: Figure S1). Algorithms were created for separating tissue compartments into stroma and epithelium, and to identify nuclei to accurately assign associations for positive staining to a specific compartment in the tumor microenvironment. To create each algorithm, 10 % of the image dataset was used for each experiment.

### Masson’s trichrome and color segmentation software

To assess collagen deposition in the tumor tissue and mammary glands, Masson trichrome staining (Cancer Diagnostics Inc., SS1026-MAB-250) was used on paraffin-embedded sections. Color images were analyzed with FIJI software and the color segmentation plugin (Daniel Sage, 2008, http://bigwww.epfl.ch/sage/soft/colorsegmentation/) using the *K*-means algorithm clustering method. All images had the same pixel size and so the total area of collagen could be quantitated as the number of blue pixels over the total number of pixels per image.

### Cytokine array

To describe a cell signaling mechanism for collagen density changes in response to high COX-2 levels and to COX-2 inhibition, a mouse cytokine ELISA plate array (Signosis, Sunnyvale, CA, USA) was used. In this quantitative chemiluminescence plate array, 23 mouse cytokines were monitored simultaneously for their expression levels in relation to collagen deposition and COX-2 inhibition with celecoxib. The cytokine signal was measured with a fluorometer (Fluoroskan, Ascent, FL) and Ascent software version 2.6 (Thermo Scientific). To compare fold-change differences, data were normalized to a blank and graphically represented by normalization to PyMT vehicle cytokine data levels.

### Positron emission tomography (PET) imaging and analysis

Highly sensitive and quantitative PET imaging was used to study potential preventative effects of COX-2 inhibition. All mice were fasted for 8 h prior to intravenous injection of approximately 5 MBq of 2′-deoxy-2′-[^18^F]fluoro-d-glucose (FDG) 1 h before imaging. Mice were anesthetized with inhalation gas (2 % isoflurane gas mixed with 1 L/minute of pure oxygen) and kept under a heat lamp during injection until imaging. Mice were imaged in a prone position on a Siemens Inveon Hybrid micro-PET/CT (Siemens Medical Solutions, Knoxville, TN, USA). A 10-minute PET scan was acquired and data were displayed as a histogram in one static frame; data were subsequently reconstructed using ordered-subset expectation maximization (OSEM) of three dimensions followed by the maximum *a posteriori* algorithm (matrix size = 128, 128, and 159; pixel size = 0.776, 0.776, and 0.796 mm; iterations = 18; subsets = 16; and beta smoothing factor = 0.004). Data were not corrected for attenuation or scatter. PET analysis was performed using Siemens Inveon software (Siemens Medical Solutions). The data were normalized to animal weight, amount of injected PET tracer, and tracer decay. A sphere was drawn and positioned over identified tumors and tumor volume and mean FDG uptake were calculated by the software.

### Statistical analysis

The analyses were performed to study the effect of COX-2 in mammary tumor progression in response to cell matrix density. Mixed linear models were used to assess differences between the various factors. The data were tested for normality and were log transformed as necessary. Every statistical test was two-sided, and a *p* value <0.05 was considered statistically significant. All analyses were performed using the procedure PROC MIXED from the SAS/STAT® software (version 9.4).

## Results

### COX-2 expression levels are elevated in collagen-dense tumors

To assess whether COX-2 is involved in tumor growth and enhanced in a collagen-dense tumor microenvironment, we used our previously characterized transgenic mouse model of increased stromal collagen based on the Col1a1^*tm1jae*^ mouse. This transgenic line has a mutation in the collagenase cleavage site of the α1 chain of collagen I, leading to increased collagen accumulation [10]. Mammary tumors were induced by the expression of the robust transgene, MMTV-PyMT, in which mammary carcinomas are driven by expression of the polyoma middle-T antigen, resulting in a mammary carcinoma that shares many histopathologic features with progression of human breast cancer [35, 36]. The resulting female progeny of Col1a1/+ x MMTV-PyMT carry the MMTV-PyMT transgene on either the WT or Col1a1^*tm1jae*^/+ background.

**Fig. 1:**
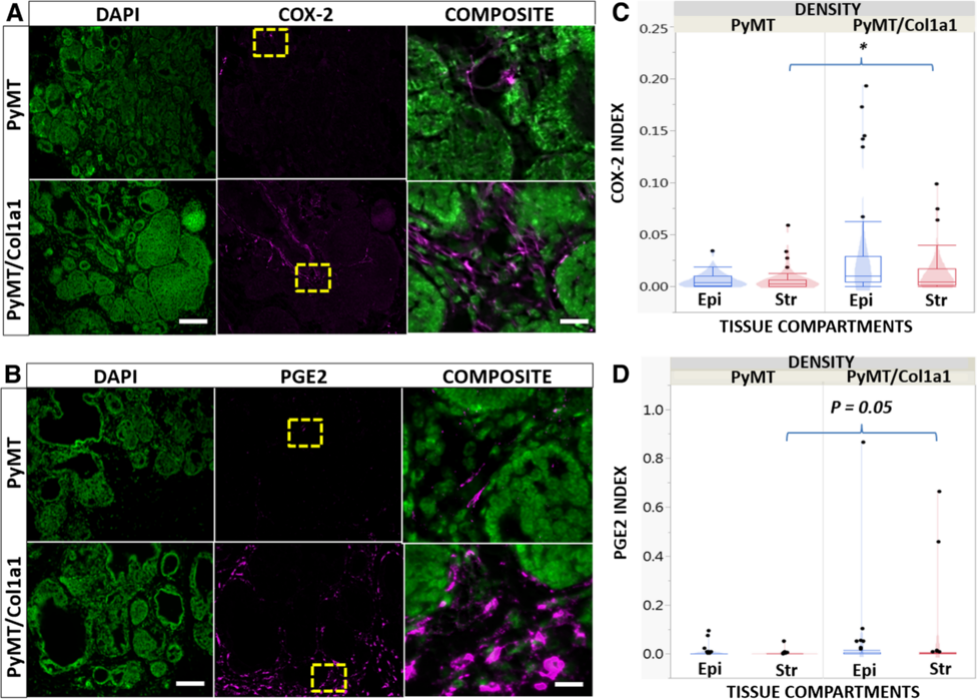
Cyclooxygenase-2 (*COX-2*) and prostaglandin E2 (*PGE2*) levels are elevated in collagen-dense tumors. **a**, **b** Immunofluorescence images of COX-2 or PGE2 (*magenta*) counterstained with 4',6-diamidino-2-phenylindole (DAPI) (*green*); ×20 objective, *scale bar* = 100 um. *Composite* is zoomed-in image of area demarked by the *yellow window*; *scale bar* = 15 um. **c**, **d** Quantitation of several images as shown in **a** and **b**. Box plots are overlaid with violin plots to indicate differences in the density distribution of data points. Raw data are depicted. *Epi* epithelium, *Str* stroma. Quantitative values represent the number of positive stained cells divided by the total number of cells in that compartment. **c** COX-2 levels are elevated in tumor and stromal cells from collagen-dense tumors in PyMT/Col1a1 compared to tumors arising in PyMT mice. **d** PGE2 levels are moderately elevated in PyMT/Col1a1 tumors in cells in the stromal compartment; *p < 0.05; n = 5 mice; at least 8 image fields analyzed per 2–3 tumors per animal

Tissue from 14-week-old nulliparous female mice was used for quantitative IF to detect COX-2 and its product, PGE2, to assess expression levels with respect to mammary tumor collagen density. We measured both COX-2 and PGE2 in the stroma adjacent to tumors and epithelium from tumors to see if their expression predominates within a particular tissue compartment. There was higher stromal COX-2 expression in the PyMT/Col1a1^*tm1jae*^ mouse mammary tumors compared to the PyMT tumors (Fig. 1a and c). Similarly, in collagen-dense tumors there was a small increase in stromal PGE2 expression (Fig. 1b and d). In the absence of tumors, we did not see a significant difference in COX-2 and PGE2 in mammary glands from PyMT mice compared to Col1a1 mice (not shown).

### COX-2 inhibition with celecoxib diminishes tumor growth and progression to metastasis

**Fig. 2:**
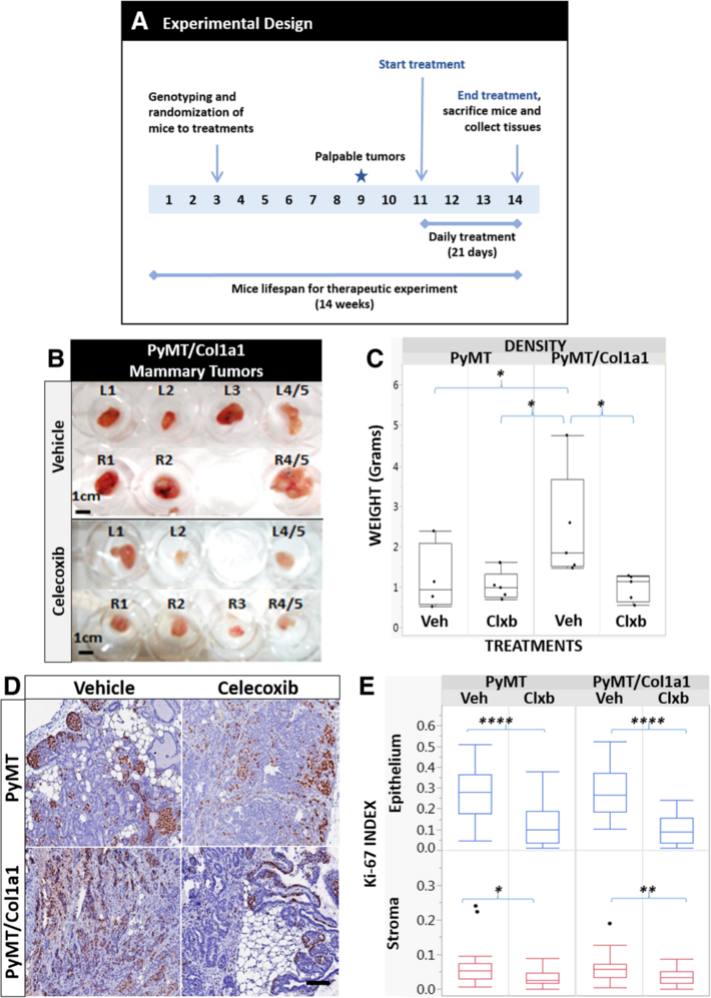
Celecoxib diminishes PyMT/Col1a1, but not PyMT, tumor growth. **a** Timeline for the celecoxib therapeutic study. See “Methods” for more details. **b** Representative example of tumors arising in collagen-dense mice treated with vehicle or celecoxib. **c** Quantitation of several tumors from mice treated with vehicle or celecoxib. Tumor weight is higher in PyMT/Col1a1 tumors when compared to PyMT tumors. Celecoxib delays tumor growth in mice with collagen-dense tumors. **d** Immunohistochemical analysis (IHC) of representative tumor sections stained with antibody against Ki-67 (visualized with 3,3-diaminobenzidine (DAB) (*brown*)) and counterstained with hematoxylin; ×20 objective; *scale bar* = 100 um. **e** Quantitation of the number of Ki67-positive cells normalized to the total number of cells. Celecoxib treatment diminishes proliferation levels as measured with the Ki-67 marker in both PyMT and PyMT/Col1a1 tumors. *Veh* vehicle, *Clxb* celecoxib. Raw data depicted; *p < 0.05, **p < 0.01, ****p < 0.0001; *n* = 5 mice; at least 8 image fields analyzed per 2–3 tumors per animal

To test whether COX-2 inhibition reverses COX-2 and PGE2 expression, we treated PyMT and PyMT/Col1a1 mice with 0.2 mg celecoxib or vehicle (5 % methyl cellulose) for 21 days (Fig. 2a). This dose was selected because it is comparable to the human dose of 600 mg a day (when using a linear scale, 10 mg/kg of body weight) and no serious adverse side effects in humans have been reported at this dose for this short period. Mice were started on a daily treatment at 11 weeks of age and tissues were collected at 14 weeks of age. At the 11-week time point, tumors were established and uniformly palpable among all experimental animals. Four treatment arms were created for this study: PyMT vehicle, PyMT celecoxib, PyMT/Col1a1 vehicle, and PyMT/Col1a1 celecoxib. PyMT/Col1a1 mice had larger tumors compared to PyMT mice and COX-2 inhibition with celecoxib diminished tumor growth only in collagen-dense tumors (Fig. 2b and c). Next, we collected lung tissue to quantify lung metastasis; despite a trend toward increased metastasis in PyMT/Col1a1 mice compared to PyMT mice, there was no statistically significant difference (Additional file 2: Figure S2). As there was a significant different in tumor growth, we measured cell proliferation in these mouse mammary tumors. IHC for detection of the proliferation marker, Ki-67, demonstrated that celecoxib diminished proliferation both in PyMT and PyMT/Col1a1 mammary tumors (Fig. 2d and e). While COX-2 levels were significantly increased in PyMT/Col1a1 tumors compared to WT tumors, COX-2 expression was significantly decreased in PyMT and high-density collagen mouse tumors when treated with celecoxib (Additional file 3: Figure S3 A-B). Moreover, PGE2 expression was significantly elevated in collagen-dense tumors and inhibition with celecoxib reversed this effect in both PyMT and PyMT/Col1a1 tumors. This suggests that COX-2 has a role in tumor growth and progression and that its inhibition has differential effects in mammary tumors arising in dense collagen compared to the WT milieu.

### Inflammatory cytokines regulated by density and COX-2 inhibition

**Fig. 3:**
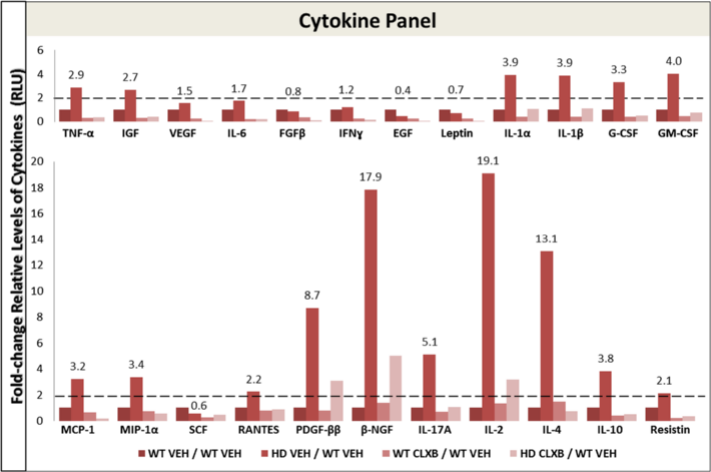
Regulation of cytokines by celecoxib in PyMT and PyMT/Col1a1 tumors. Several cytokines are upregulated in PyMT/Col1a1 (*HD*) mammary tumors compared to PyMT (wild-type (*WT*)) tumors. Treatment with celecoxib (*CLXB*) diminishes cytokine levels in PyMT (WT) and PyMT/Col1a1 (HD) mice. Relative levels of cytokines are represented as the fold-change normalized to PyMT (WT) vehicle (*VEH*). Cytokines with equal to or greater than a two-fold change are considered significantly upregulated. Three tumors, each from a single animal, were pooled per treatment arm to perform the multiplex cytokine ELISA array. *TNFα* tumor necrosis factor alpha, *IGF* insulin growth factor, *VEGF* vascular endothelial growth factor, *IL* interleukin, *FGFβ* fibroblast growth factor beta, *IFNy* interferon gamma, *EGF* epithelial growth factor, *G-CSF* granulocyte-colony stimulating factor (CSF-2), *GM-CSF* granulocyte-macrophage colony-stimulating factor (CSF-3), *MCP-1* monocyte chemotactic protein 1 (CCL2), *MIP-1α* macrophage inflammatory protein 1 alpha (CCL3), *SCF* stem cell factor, *RANTES* regulated on activation, normal T cell expressed and secreted (CCL5), *PDGF-ββ* platelet-derived growth factor beta-beta, *β-NGF* nerve growth factor beta

Cancer, inflammatory and stromal cells can secrete and respond to cytokines that stimulate growth, diminish apoptosis, and enable invasion and metastasis in the tumor microenvironment. Having demonstrated that dense collagen tumors have increased expression of COX-2, and that its over-expression enhanced tumor growth, cell proliferation, and metastasis, we investigated the role of COX-2 in inflammation within collagen-dense tumor microenvironments using a quantitative chemiluminescence assay to detect the expression of 23 cytokines. Samples from three mouse tumors for each of the study arms were pooled and cytokines were monitored simultaneously for their expression relative to collagen deposition and COX-2 inhibition with celecoxib. Most cytokines were increased two-fold or more in PyMT/Col1a1 tumors compared to PyMT tumors. Cytokines with substantially increased expression included IL-2 (19-fold), β- NGF (17-fold), IL-4 (13-fold), platelet-derived growth factor (PDGF) (8.7-fold) and IL-17A (5-fold) (Fig. 3). COX-2 inhibition with celecoxib diminished overall cytokine expression levels in both PyMT and PyMT/Col1a1 mice (Fig. 3). These results indicate that there is an effect of high collagen density in altering cytokine expression levels, and that these high cytokine expression levels are decreased by COX-2 inhibition with celecoxib.

**Fig. 4:**
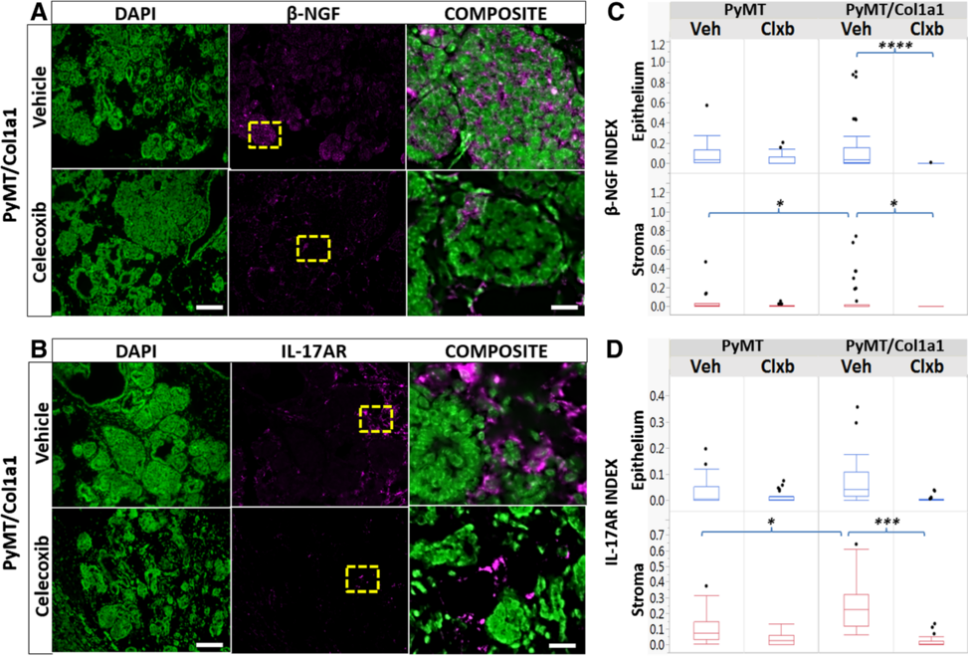
Immunofluorescence (IF) validation of cytokine data in tumor and stroma tissue. **a**, **b** Representative IF images of nerve growth factor beta (*β-NGF*) or IL-17A receptor (*IL-17A-R*) (*magenta*) counterstained with 4',6-diamidino-2-phenylindole (*DAPI*) (*green*); ×20 objective; *scale bar* = 100 um. *Composite* is an enlarged image of the area demarked by the *yellow window*; *scale bar* = 15 um. **c** Quantitation of β-NGF normalized to the total number of cells. β-NGF is elevated in the stroma of PyMT/Col1a1 tumors. Celecoxib (*Clxb*) decreases β-NGF in both the epithelium and stroma in PyMT/Col1a1 tumors. β-NGF is higher in the tumor epithelium, consistent with its known role in promoting survival and proliferation of epithelial breast cancer cells. **d** Quantitation of IL-17A-R normalized to the total number of cells. IL-17AR is elevated in the stroma of PyMT/Col1a1 tumors, consistent with its role in macrophage/neutrophil recruitment. Celecoxib treatment decreases IL-17A-R only in the stroma of PyMT/Col1a1 tumors. Raw data are depicted; *p < 0.05, ***p < 0.001, ****p < 0.0001; *n* = 5 mice per arm; at least 8 image fields analyzed per 2–3 tumors per animal; mixed linear model. *Veh* vehicle

To validate the cytokine expression in the epithelium and stroma of the tumors, we performed quantitative IF for two highly expressed cytokines. We studied overall β-NGF distribution in the tumor microenvironment. β-NGF is secreted by epithelial cells, macrophages, neutrophils, and neurons. It is expressed in 80 % of breast cancers and it activates the survival and proliferation of tumor cells [37, 38]. Overall, we found that β-NGF expression was higher in the tumor epithelial compartment than in the stroma, and that there was more stromal β-NGF in PyMT/Col1a1 tumors compared to PyMT tumors. Treatment with celecoxib reversed β-NGF in PyMT/Col1a1 tumors, within both the epithelial and stromal compartments (Fig. 4a and c).

Because we observed high levels of IL17A in the cytokine array data, we characterized the expression of IL-17A receptor (IL-17A-R) as a measure of cell populations that may be recruited by this cytokine. We observed that IL-17A-R is mostly expressed by cell populations in the stromal compartment, and was significantly elevated in PyMT/Col1a1 tumors compared to PyMT tumors (Fig. 4b and d). Celecoxib treatment inhibited IL-17A-R-expressing cell populations within the stroma of the collagen-dense tumor microenvironment (Fig. 4b and d). Together these findings indicate that tumor density and COX-2 expression play a role in cytokine expression, perhaps via inflammatory pathways.

### Collagen-dense tumors and COX-2 regulate macrophage and neutrophil populations

**Fig. 5:**
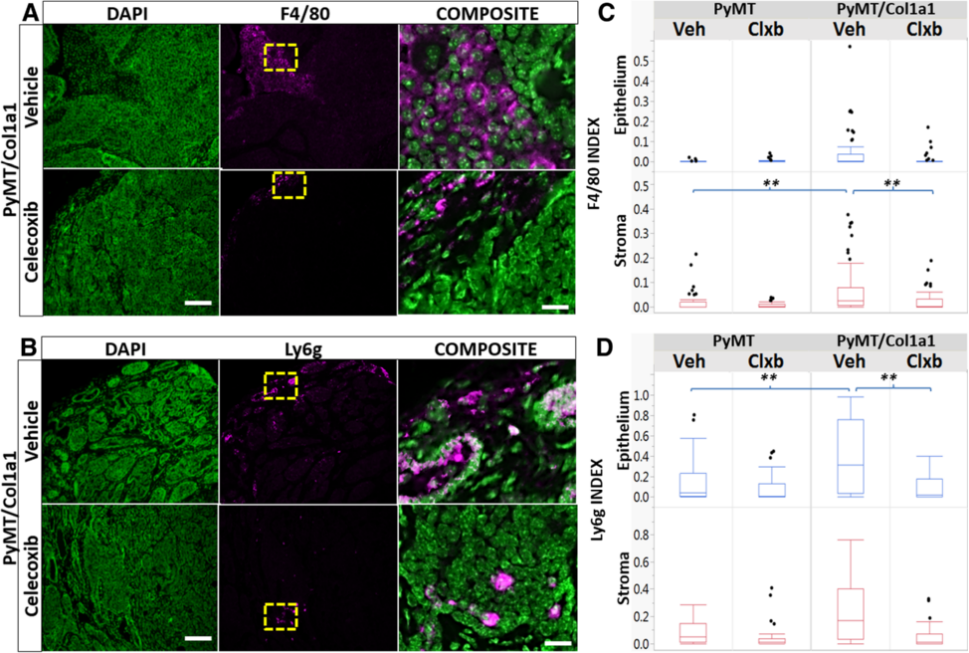
Regulation of macrophages and neutrophils by collagen density and celecoxib. **a**, **b** Immunofluorescence images of F4/80^+^ macrophages or Ly6g^+^ neutrophils (*magenta*), respectively, counterstained with 4',6-diamidino-2-phenylindole (*DAPI*) (*green*); ×20 objective; *scale bar* = 100 um. **c**, **d** Index was calculated by dividing positive-stained cells by total numbers of cells. Raw data are depicted. **c** Quantitation of total F4/80^+^ cells normalized to the total number of cells. F4/80^+^ macrophage numbers are elevated in the stroma of PyMT/Col1a1 tumors and decreased by treatment with celecoxib. **d** Total number of Ly6g^+^ neutrophils normalized to total number of cells. Ly6g^+^ neutrophil numbers are elevated in the epithelium of PyMT/Col1a1 tumors and decreased by treatment with celecoxib (*Clxb*); **p < 0.01; *n* = 5 mice per arm; at least 8 image fields analyzed per 2–3 tumors per animal; mixed linear model. *Veh* vehicle

To characterize cytokine-mediated recruitment of inflammatory and stromal cell populations to the tumor microenvironment, we performed quantitative IF to identify populations of mature macrophages (F4/80-positive) and neutrophil granulocytes (Ly6g-positive). Stromal F4/80 macrophages were significantly increased in PyMT/Col1a1 tumors compared to PyMT tumors. Treatment with celecoxib diminished F4/80^+^ macrophage numbers in collagen-dense tumors (Fig. 5a and c). Moreover, epithelial Ly6g^+^ neutrophils were also increased in the collagen-dense tumors compared to PyMT tumors and celecoxib diminished Ly6g^+^ neutrophil populations only in PyMT/Col1a1 tumors as well (Fig. 5b and d). These results reinforce the data from our cytokine array study and suggest that increased tumor collagen density leads to increased COX-2 function and recruits macrophages and neutrophils into the collagen-dense microenvironment.

### Collagen-dense tumors and COX-2 regulate fibroblast populations

**Fig. 6:**
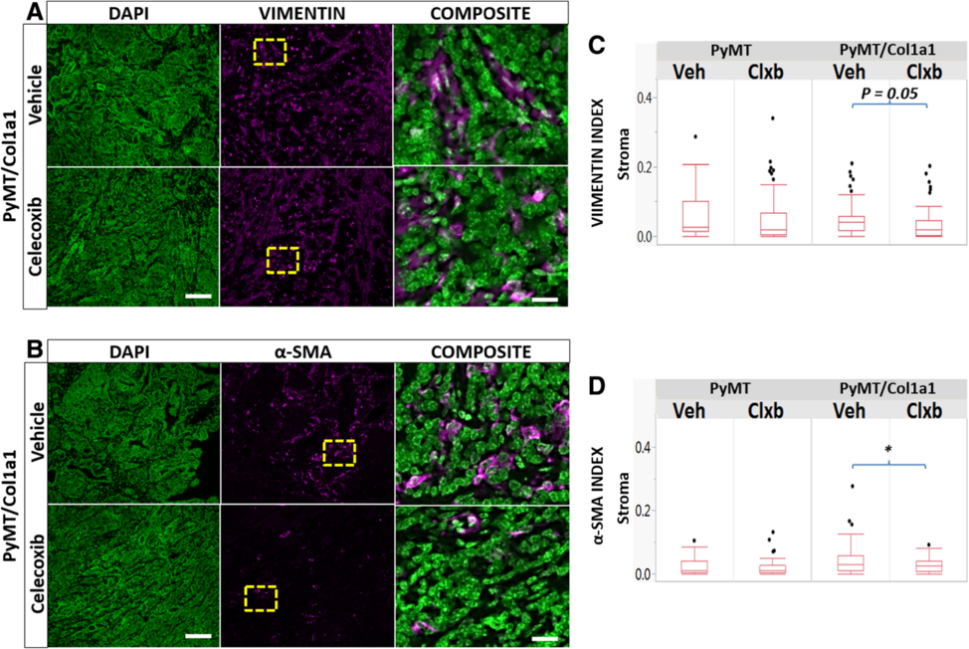
Regulation of fibroblasts by collagen density and celecoxib. **a**, **b** Immunofluorescence images of vimentin^+^ fibroblasts or alpha smooth muscle actin (*α-SMA*)^+^ fibroblasts (*magenta*) counterstained with 4',6-diamidino-2-phenylindole (*DAPI*) (*green*); ×20 objective; *scale bar* = 100 um. **c** The number of vimentin^+^ fibroblasts remains the same regardless of collagen density. Values represent vimentin^+^ cells normalized to total cell number. **d** Celecoxib (*Clxb*) decreases the number of α-SMA^+^ fibroblasts in the stroma of PyMT/Col1a1 tumors. Values represent the number of α-SMA^+^ cells normalized to the total number of cells. Raw data are depicted; *p < 0.05; n = 5 mice per arm; at least 8 image fields analyzed per 2–3 tumors per animal; mixed linear model. *Veh* vehicle

Our cytokine array data revealed elevation of several cytokines and growth factors associated with tumor cell proliferation and inflammatory response modulation in the PyMT/Col1a1 tumor microenvironment (Fig. 3). To determine whether the collagen-dense microenvironment and high COX-2 levels regulate different fibroblast populations known to secrete such factors, we performed quantitative IF with vimentin as a general fibroblast marker and α-SMA as a marker of cancer-associated fibroblasts (CAF). We observed that stromal vimentin^+^ fibroblast populations were similar in all treatment arms of the study. However, there was a trend toward decreased vimentin^+^ fibroblast numbers only in the PyMT/Col1a1 tumors with celecoxib treatment (Fig. 6a and c). Notably, celecoxib decreased α-SMA^+^ fibroblasts within the PyMT/Col1a1 tumor microenvironment (Fig. 6b and d). Few cells that are vimentin-positive or aSMA-positive co-stain for CD31, indicating that the cells we originally identified as CAF are not microvasculature (not shown). Nor did the number of CD31+ cells account for the effect of celecoxib on tumor regression under high density conditions (not shown).

**Fig. 7:**
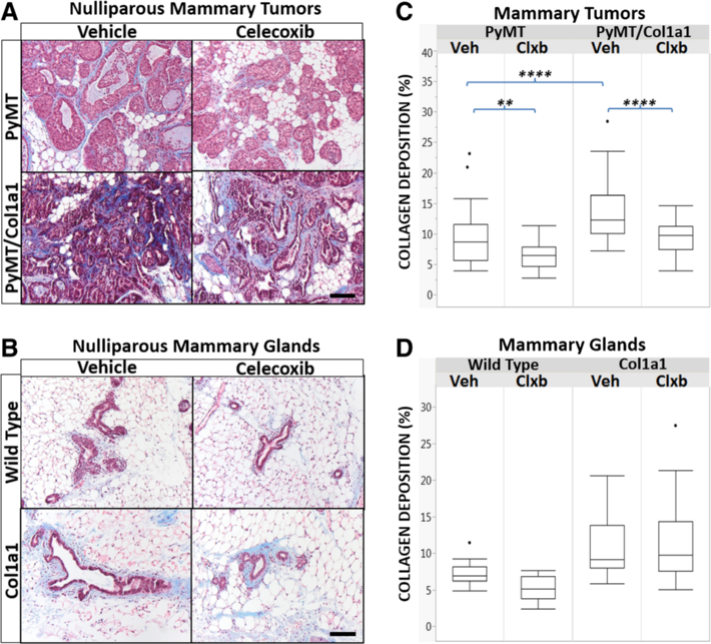
Celecoxib diminishes collagen deposition in PyMT mammary tumors, but does not affect tumor-free mammary glands. **a**, **b** Masson’s trichrome stain of mammary tumors or mammary glands of nulliparous mice treated with vehicle or celecoxib. Collagen fibers are in *blue*, cell nuclei are *black* and the cell cytoplasm, muscle tissue, and erythrocytes are stained *red*; ×20 objective; *scale bar* = 100 um. **c**, **d** Quantitation of Masson’s trichrome, performed as described in “Methods”. **a**, **c** There is significantly increased collagen in PyMT/Col1a1 compared to PyMT tumors. Celecoxib (*Clxb*) diminishes collagen deposition in both PyMT and PyMT/Col1a1 tumors. **b**, **d** There is no significant effect of treatment with celecoxib in normal, tumor-free mammary glands. Glands in Col1a1 animals tend to have greater collagen accumulation than their wild-type counterparts. Raw data are depicted; **p < 0.01, *p* < 0.0001; *n* = 5 mice per arm for **a** and **c** and *n* = 1 mouse per arm for **b** and **d**; **a **and **b** are separate animal cohorts, age is 14 weeks for both animal cohorts; at least 5 image fields analyzed per 2–3 tumors/glands per animal; mixed linear model. *Veh* vehicle

As CAF induce higher collagen deposition to alter the extracellular matrix, and COX-2 inhibition diminishes α-SMA^+^ fibroblasts in PyMT/Col1a1 tumors, we characterized collagen levels in mammary tumors using Masson’s trichrome staining. There was more collagen deposited in PyMT/Col1a1 tumors and COX-2 inhibition by celecoxib-reversed collagen levels in both PyMT and PyMT/Col1a1 tumors (Fig. 7a and c). To discriminate effects related to tumor formation vs COX-2 treatment, we stained the mammary glands of nulliparous mice treated with celecoxib or vehicle in PyMT and PyMT/Col1a1 mice. COX-2 inhibition by celecoxib did not affect collagen deposition in the mammary glands of either PyMT or PyMT/Col1a1 mice (Fig. 7b and d). These results indicate that inhibition of COX-2 by celecoxib specifically affects the tumors of PyMT/Col1a1 mice by reducing CAF populations, and by diminishing the tumor-associated collagen deposition prevalent in PyMT/Col1a1 tumors.

### Administration of celecoxib prior to tumor formation diminishes tumor growth and number

**Fig. 8:**
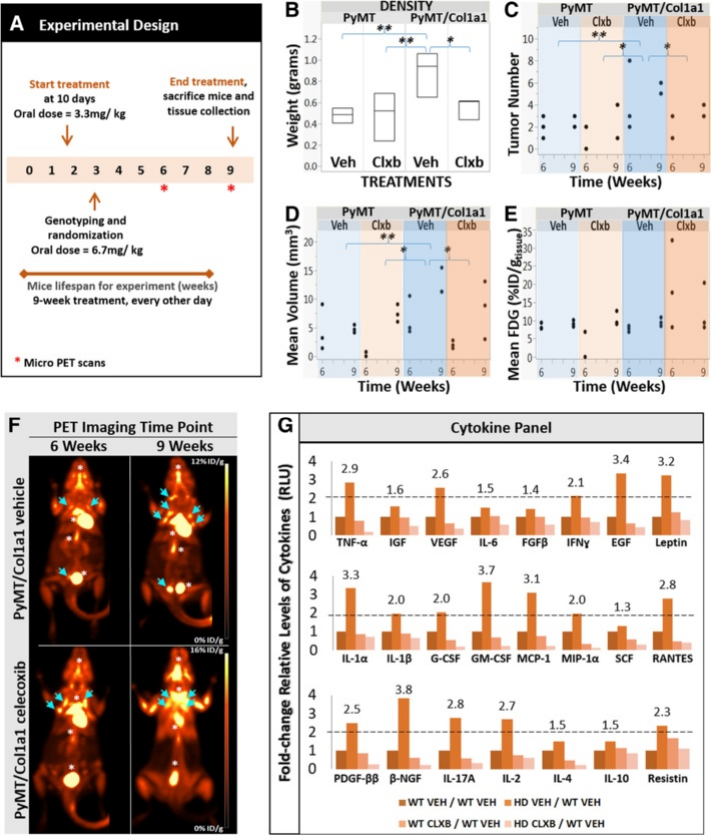
Celecoxib diminishes PyMT/Col1a1 tumor growth when administered prior to tumor formation. **a** Timeline for the celecoxib preventive study. See “Methods” for more details. **b**-**e** Quantitation of several tumors from PyMT and PyMT/Col1a1 animals, treated with vehicle (*Veh*) or celecoxib (*Clxb*). **b** Tumor weight is higher in PyMT/Col1a1 tumors (n = 5) when compared to PyMT tumors and celecoxib delays tumor growth in PyMT/Col1a1 mice. **c** PyMT/Col1a1 mice develop more tumors than PyMT mice and celecoxib reduces tumor number in PyMT/Col1a1 mice. **d** Tumor volume is higher in PyMT/Col1a1 tumors when compared to PyMT tumors and celecoxib reduces tumor volume in PyMT/Col1a1 mice. **e** The average amount of glucose uptake by the tumors remains the same regardless of collagen density or treatment with celecoxib. ^18^Fluorodeoxyglucose (FDG) positron emission tomography (*PET*) tracer. *%ID/g*_*(tissue)*_ is the percent injected dose of PET tracer per gram of tissue. **f** Representative PET images of PyMT/Col1a1 mice at 9 and 14 weeks of age either treated with celecoxib or vehicle. Images over time correspond to same subject. *Arrows* indicate tumors and *asterisks* indicate tissue other than tumors that uptake the FDG tracer, such as brain, carotid, brown fat, heart, kidneys, aorta, bladder, or muscle tissue. **g** Regulation of cytokines by density and celecoxib. Several cytokines are upregulated in PyMT/Col1a1 (HD) mammary tumors compared to PyMT (wild-type (*WT*)) tumors. Treatment with celecoxib diminishes cytokine levels in PyMT (WT) and PyMT/Col1a1 (HD) mice. Relative levels of cytokines are represented as the fold-change normalized to PyMT (WT) vehicle. Cytokines with equal to or greater than a 2-fold change are considered significantly upregulated. Three tumors, each from a single animal, were pooled for each treatment arm to perform the multiplex cytokine ELISA array. *TNFa* tumor necrosis factor alpha, *IGF* insulin growth factor, *VEGF* vascular endothelial growth factor, *IL* interleukin, *FGFβ* fibroblast growth factor beta, *IFNy* interferon gamma, *EGF* epithelial growth factor, *G-CSF* granulocyte-colony stimulating factor (CSF-2), *GM-CSF* granulocyte-macrophage colony-stimulating factor (CSF-3), *MCP-1* monocyte chemotactic protein 1 (CCL2), *MIP-1a* macrophage inflammatory protein 1 alpha (CCL3), *SCF* stem cell factor, *RANTES* regulated on activation, normal T cell expressed and secreted (CCL5), *PDGF-ββ* Platelet-derived growth factor beta-beta, *β*-*NGF* nerve growth factor beta. **c**-**f** For the PET studies, *n* = 3 and for tumor weight measurement, *n* = 5 mice; *p < 0.05, **p < 0.01

To study whether celecoxib inhibition of COX-2 expression in response to collagen density might be effective as a preventive breast cancer therapy, we treated early postnatal animals before palpable tumors arose. In the MMTV-PyMT model, tumors arise at puberty, when the mammary gland develops. Female PyMT/Col1a1 and their WT PyMT counterparts at 10 days of age (well before puberty) were randomly assigned to treatment with celecoxib or vehicle (Fig. 8a). Mice were treated until they were 9 weeks of age and their tissues were collected for analysis. PET scans were used to determine mammary gland tumor weight, number, growth rate, and volume in PyMT and PyMT/Col1a1 mice with and without celecoxib treatment. Mice were imaged at 6 weeks and 9 weeks. At 9 weeks of age, mice bearing collagen-dense tumors had larger tumors when compared to PyMT tumors. Consistent with the above treatment regimen, celecoxib diminished tumor weight only in PyMT/Col1a1 mice (Fig. 8b). Tumor growth features were measured and compared longitudinally over time using PET scans, and the number of tumors was counted at 6 and 9 weeks of age. Histopathological analysis at 9 weeks of age confirmed that each tumor assessed by PET was indeed a tumor, and not otherwise hypermetabolic tissue (Additional file 4: Figure S4). Over time the collagen-dense mice developed more tumors than PyMT mice. Celecoxib reduced tumor numbers only in PyMT/Col1a1 mice (Fig. 8c and f). In addition, the rate of increase in tumor volume was enhanced over time in mice bearing PyMT/Col1a1 tumors compared to PyMT tumors. Again, celecoxib reduced tumor volume in PyMT/Col1a1 mice (Fig. 8d). Surprisingly, we did not find differences when we measured mean glucose uptake with ^18^Fluorodeoxyglucose-PET tracer, suggesting that neither density nor treatment with celecoxib altered glucose metabolism (Fig. 8e).

To further understand the underlying mechanisms of COX-2 inhibition and its response to the collagen-dense tumor microenvironment in this early treatment setting, we measured levels of different cytokines using the multiplex ELISA cytokine array described above. The relative levels of cytokines followed the same trends as in the later therapeutic study (Fig. 3). Most cytokines had 2-fold increased expression: the greatest increases were in the expression of epithelial growth factor (EGF) (3.4-fold), leptin (3.2-fold), IL-1α (3.3-fold), granulocyte macrophage colony stimulating factor (GM-CSF) (3.7-fold), monocyte chemotactic protein 1 (MCP-1) (3.1-fold), and β-NGF (3.8-fold) (Fig. 8g). Collagen-dense tumors tended to express significantly higher levels of cytokines compared to WT tumors. Additionally, when PyMT and PyMT/Col1a1 mice were treated with celecoxib, the relative levels of cytokines declined (Fig. 8g). Similar to the later therapeutic study, these results suggest that collagen density affects expression of cytokines in a manner that is reversed by celecoxib. Together, these data indicate that COX-2 is a significant driver of tumor formation and growth in collagen-dense tumors. Celecoxib reverses the increased tumor progression of the collagen-dense microenvironment, although it cannot completely prevent tumor incidence in this genetically driven MMTV-PyMT mouse model.

## Discussion

COX-2 over-expression in breast cancer is associated with poor patient prognosis and is emerging as a key mediator of tumor inflammation. Here, we determined the role of COX-2 in regulating the enhanced tumor formation and progression that we observed in the collagen-dense tumor microenvironment. We found that PyMT/Col1a1 tumors arising in collagen-dense tissue were associated with a more inflammatory microenvironment than PyMT tumors arising in WT mice. Over-expression of COX-2 is a major contributor to the inflammatory milieu of collagen-dense tumors, and leads to recruitment of tumor-associated macrophages (TAMs) and tumor-associated neutrophils (TANs). Subsequently, we demonstrated that we can reverse recruitment of inflammatory cell populations in the dense tumor microenvironment by selectively inhibiting COX-2 with celecoxib. In addition, celecoxib reduced collagen deposition and decreased αSMA^+^ fibroblasts, mainly in the PyMT/Col1a1 tumors. These data suggest there may be a therapeutic opportunity to treat tumors arising in dense breast tissue with celecoxib. As the clinical findings with celecoxib as a therapeutic or preventative approach to breast cancer have been mixed, our data suggest that selection of the right patients - those with dense breast tissue - may result in the appropriate context by which treatment with celecoxib might be more effective.

Consistent with the enhanced inflammatory environment of the collagen-dense tumors, we observed a dramatic increase in several cytokines in the PyMT/Col1a1 tumors. Several granulocyte and macrophage-recruiting cytokines, including GM-CSF, G-CSF, MCP-1, MIP-1α and RANTES (CCL5), were increased in collagen-dense tumors. Moreover, there was a strong increase in IL-17A, which contributes to macrophage and neutrophil recruitment [39]. IL-17A is a pro-inflammatory cytokine that is secreted by T helper (Th)17 cells and induces the production of other cytokines, growth factors, and prostaglandins from other cells, including fibroblasts [39]. Also, IL-17A promotes angiogenesis, cell proliferation, and chemoresistance and it is associated with poor patient prognosis [40, 41]. We observed increased levels of IL-17R-A in the stroma of collagen-dense tumors, and these levels decreased after celecoxib treatment. Accordingly, decline of IL17A and its receptor expression levels after treatment with celecoxib was accompanied by declining populations of F4/80^+^ macrophages and Ly6g^+^ neutrophils. Recent findings demonstrate a role for COX-2 in the immune checkpoint, allowing melanomas to escape immune surveillance [42]. Based on our findings that the collagen-dense microenvironment increases COX-2 and regulates inflammation, it is possible that a collagen-dense microenvironment will have a role in regulating the immune checkpoint as well.

PDGF-ββ is produced by epithelial and endothelial cells and stimulates nearby mesenchymal cells, including fibroblasts, in a paracrine fashion [43]. Consistent with this, we observed significant over-expression of PDGF-ββ in PyMT/Col1a1 tumors compared to PyMT tumors, and COX-2 inhibition by celecoxib diminished this effect. COX-2 inhibition with celecoxib decreased αSMA^+^ fibroblasts in only the PyMT/Col1a1 tumors, even though this population was not significantly increased in the PyMT/Col1a1 tumors. CAF secrete elevated levels of many growth factors, including β-NGF. The continuous secretion of β-NGF activates an autocrine loop whereby more fibroblasts are recruited [44]. Additionally, β-NGF can be secreted by epithelial cells, macrophages, and neutrophils. Interestingly, we found that stromal β-NGF is elevated only in PyMT/Col1a1 tumors and COX-2 inhibition with celecoxib decreases β-NGF over-expression in both PyMT and collagen-dense tumors.

COX-2 also regulates increased collagen deposition. Treatment with celecoxib leads to a reversal of increased collagen deposition that occurs around tumors, even in the PyMT/Col1a1 tumors. This finding adds important insight into the mechanism of desmoplasia often observed around breast tumors. Moreover, these data are consistent with our observation that celecoxib diminished matrix deposition. Interestingly, Lyons et al. also demonstrated that COX-2 inhibition reduces the collagen deposition associated with tumor growth and progression to metastasis in the involuting mammary gland [12]. Our results suggest that greater collagen deposition and elevated levels of COX-2 promote activation of more αSMA^+^ fibroblasts, which in turn elicit greater deposition of collagenous stroma. Moreover, it has been demonstrated that macrophages are associated with local regions of collagen deposition in the postpartum involuting mammary gland [45], suggesting that increased recruitment of macrophages could also contribute to elevated collagen deposition in PyMT/Col1a1 tumors. Consistent with this, we observed reduced numbers of CAF and F4/80^+^ macrophages when collagen-dense tumors were treated with celecoxib. These data suggest a feed-forward mechanism by which a collagen-dense microenvironment leads to greater COX-2 expression, increased inflammatory cells and CAF, and in turn, additional increased stromal deposition.

Celecoxib, with its anti-inflammatory effects, is associated with less risk of endoscopic mucosal injury [46] and decreased incidence of cardio-renal toxicity [31] compared with ibuprofen. There is evidence that COX-2 inhibitors can decrease breast cancer risk by 16 % [47]. Thus, we tested whether celecoxib might be a candidate to consider for chemoprevention in women with dense breast tissue. Using no more than the maximum celecoxib dosage recommended by the FDA, we showed that COX-2 inhibition with celecoxib results in smaller and fewer tumors than treatment with vehicle alone in PyMT/Col1a1 mice. In addition, in PyMT/Col1a1 mice, treatment with celecoxib decreased expression of all 23 cytokines tested in our ELISA array. This indicates that COX-2 inhibition modulates several immune and stromal cell populations and delays tumor formation and progression. We recognize that this is not a true chemoprevention preclinical finding, as a major limitation of the PyMT mouse model is that it is a very aggressive genetically-driven model of mammary carcinoma. Thus, it would have been impossible to observe complete prevention of mammary tumors in this context.

There is evidence of the effect of celecoxib as a therapeutic agent for primary breast cancer. A randomized controlled phase II clinical trial demonstrated that preoperative treatment with celecoxib changes the expression of several genes at the transcription level in patients with invasive breast cancer when compared to placebo [48]. Additionally, Fabi et al. found that treatment with celecoxib facilitates the tolerability of capecitabine, a drug that aids in the delivery of the anti-cancer agent 5-fluorouracil (5-FU) in patients with metastatic breast cancer. Results from this phase II clinical trial showed that in patients with COX-2 over-expressing tumors the time to progression of disease and median overall survival was significantly longer with celecoxib treatment [49]. Moreover, the increased gene expression of both COX-2 and collagen I is associated with decreased survival and a shorter time to development of metastasis [12]. Added to these findings, our results suggest that COX-2 may be an effective preventive or therapeutic molecular target that will preferentially benefit women with dense breast tissue. A clinical trial to study the impact of celecoxib on women with dense breast tissue and high COX-2 expression would be of great clinical significance.

## Conclusions

Our findings support a mechanism by which COX-2 modulates tumor progression in collagen-dense matrices and contributes to a more aggressive tumor microenvironment. In addition, in dense mammary tumors, COX-2 inhibition with celecoxib reduces tumor formation and growth, collagen deposition, and expression of several cytokines. These findings suggest that COX-2 has a direct role in modulating tumor progression specifically in tumors arising within collagen-dense microenvironments, but minimal effects in non-dense microenvironments. As dense breast tissue is a common occurrence, and promotes more aggressive tumors, these results suggest that COX-2 may be an effective therapeutic target for women with dense breast tissue.

## Additional files

Additional file 1: Figure S1. Tissue segmentation analysis. **a**-**d** Images for the process of tissue segmentation. Algorithms for tissue segmentation, i.e., tumor epithelium versus tumor stroma, were created by machine learning (see “Methods”). **a** Sample IF image cube of mouse tumor stained with COX-2 (*green*) and counterstained with DAPI (*blue*); ×20 objective; *scale bar* = 100 um. **b** Tissue segmentation mask after training the software. *Red* epithelium, *green* stroma, *blue* other (empty space, debris/artifacts). **c** Tissue segmentation map (*overlay* of tissue segmentation mask and IF image). **d** Overlay of object cell count map and tissue segmentation map. Each object (cell) *circled in green* was associated with its respective tissue compartment; tumor epithelium or stroma and debris was associated with the “other” category and not included in the statistical analysis. (PNG 1438 kb) Additional file 2: Figure S2. COX-2 inhibition and lung metastasis. **a** Lung metastases tend to be increased in PyMT/Cola1a tumors and are inhibited by celecoxib (n.s.). *Gray shading* depicts data density graph to better illustrate differences in data distribution. *Veh* vehicle, *Clxb* celecoxib. Graphs depict raw data; *n* = 5 mice per arm. (PNG 31 kb) Additional file 3: Figure S3. Celecoxib diminishes COX2 and PGE2. **a** IHC images of COX-2 and PGE2 (DAB) counterstained with hematoxylin; ×40 objective; *scale bar* = 50 um. **b**, **c** Index was calculated by dividing amount of positive-stained cells over total amount of cells. Graphs depict raw data. **b** COX-2 levels are elevated in PyMT/Col1a1 tumors. Celecoxib diminishes COX-2 levels in PyMT and PyMT/Col1a1 tumors. **c** PGE2 levels are elevated in PyMT/Col1a1 tumors and celecoxib diminishes PGE2 levels in PyMT and PyMT/Col1a1 tumors; **p* < 0.05, ***p* < 0.01, *****p* < 0.0001; *n* = 5 mice per arm; at least 8 image fields analyzed per 2–3 tumors per animal; mixed linear model. (PNG 1241 kb) Additional file 4: Figure S4. Histology of mammary tumors after FDG-PET imaging in the early treatment study. Hematoxylin and eosin stained sections of mammary tumors from PyMT and PyMT/Col1a1 mice at 9 weeks of age, treated with celecoxib or vehicle. Histological images confirm the difference in tumor growth between PyMT and PyMT/Col1a1 mice and tumor growth reduction in PyMT/Col1a1 mice when treated with celecoxib prior to tumor formation; ×4 objective; *scale bar* = 0.5 mm. (PNG 430 kb)

## Abbreviations

5-FU: 5-fluorouracil
CAF: cancer-associated fibroblasts
COX-1: cyclooxygenase-1
COX-2: cyclooxygenase-2
DAB: 3,3-diaminobenzidine
DAPI: 4',6-diamidino-2-phenylindole
ECM: extracellular matrix
EGF: epithelial growth factor
ELISA: enzyme-linked immunosorbent assay
EMT: epithelial-mesenchymal transition
FDA: Food and Drug Administration
FDG: 2′-deoxy-2′-[^18^F]fluoro-d-glucose
FFPE: formalin-fixed paraffin embedding
FGFβ: fibroblast growth factor beta
G-CSF: granulocyte-colony stimulating factor (CSF-2)
GM-CSF: granulocyte-macrophage colony-stimulating factor (CSF-3)
HD: Col1a1^*tm1jae*^ (mice carrying the collagenase transgene)
IF: immunofluorescence
IFNγ: interferon gamma
IGF: insulin growth factor
IHC: immunohistochemical analysis
IL: interleukin
IL-17A: interleukin 17A
MCP-1: monocyte chemotactic protein 1 (CCL2)
MIP-1α: macrophage inflammatory protein 1 alpha (CCL3)
MMTV: mouse mammary tumor virus
NSAID: non-steroidal anti-inflammatory drug
OSEM: ordered-subset expectation maximization
PDGF-ββ: platelet-derived growth factor ββ
PET: positron emission tomography
PGE2: prostaglandin E2
PTGS2: prostaglandin-endoperoxide synthase 2
PyMT: polyomavirus middle T
RANTES: regulated on activation, normal T cell expressed and secreted (CCL5)
SCF: stem cell factor
TACS-3: tumor associated collagen signature-3
TAMs: tumor-associated macrophages
TANs: tumor-associated neutrophils
TNFα: tumor necrosis factor alpha
VEGF: vascular endothelial growth factor
VIM: vimentin
WT: wild-type
αSMA: alpha smooth muscle actin
β-NGF: nerve growth factor β
