# Supplementary figures and images for: COX-2 modulates mammary tumor progression in response to collagen density
Source: Breast Cancer Res. 2016 Mar 22;18:35. doi: 10.1186/s13058-016-0695-3 (PMC4802888; doi:10.1186/s13058-016-0695-3)

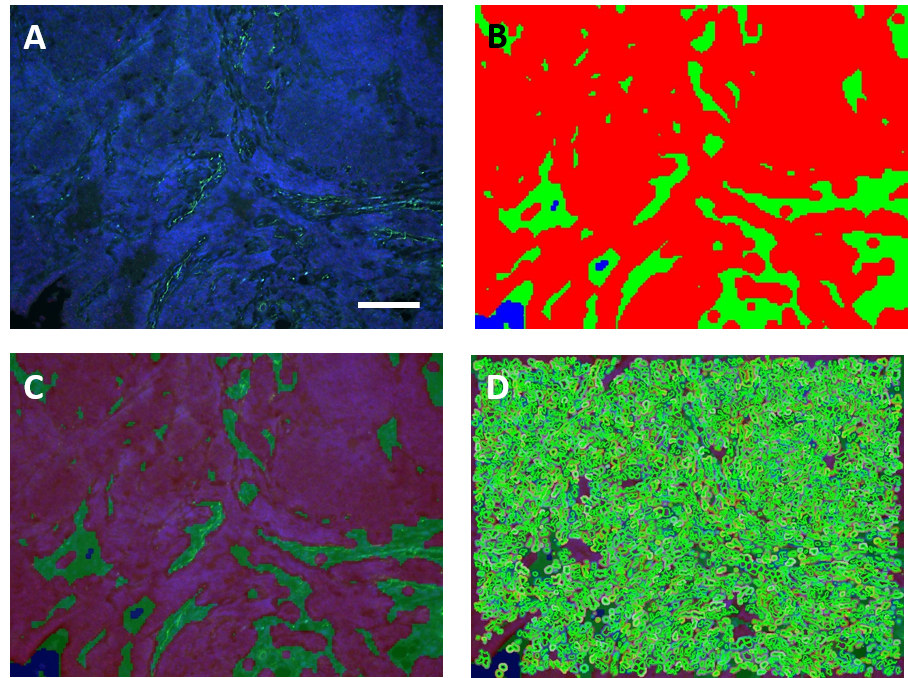

Supplement: Additional file 1: Figure S1. — Tissue segmentation analysis. a-d Images for the process of tissue segmentation. Algorithms for tissue segmentation, i.e., tumor epithelium versus tumor stroma, were created by machine learning (see “Methods”). a Sample IF image cube of mouse tumor stained with COX-2 (green) and counterstained with DAPI (blue); ×20 objective; scale bar = 100 um. b Tissue segmentation mask after training the software. Red epithelium, green stroma, blue other (empty space, debris/artifacts). c Tissue segmentation map (overlay of tissue segmentation mask and IF image). d Overlay of object cell count map and tissue segmentation map. Each object (cell) circled in green was associated with its respective tissue compartment; tumor epithelium or stroma and debris was associated with the “other” category and not included in the statistical analysis. (PNG 1438 kb) [file 13058_2016_695_MOESM1_ESM.png]

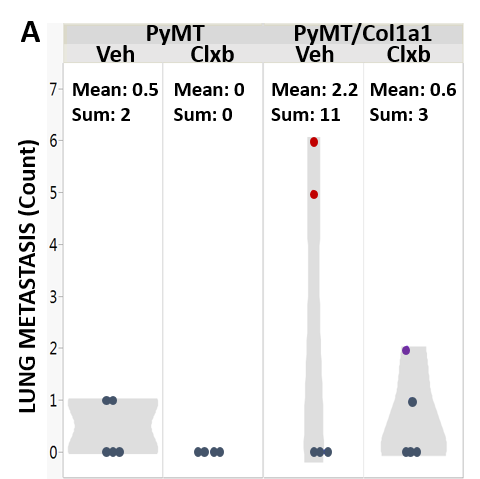

Supplement: Additional file 2: Figure S2. — COX-2 inhibition and lung metastasis. a Lung metastases tend to be increased in PyMT/Cola1a tumors and are inhibited by celecoxib (n.s.). Gray shading depicts data density graph to better illustrate differences in data distribution. Veh vehicle, Clxb celecoxib. Graphs depict raw data; n = 5 mice per arm. (PNG 31 kb) [file 13058_2016_695_MOESM2_ESM.png]

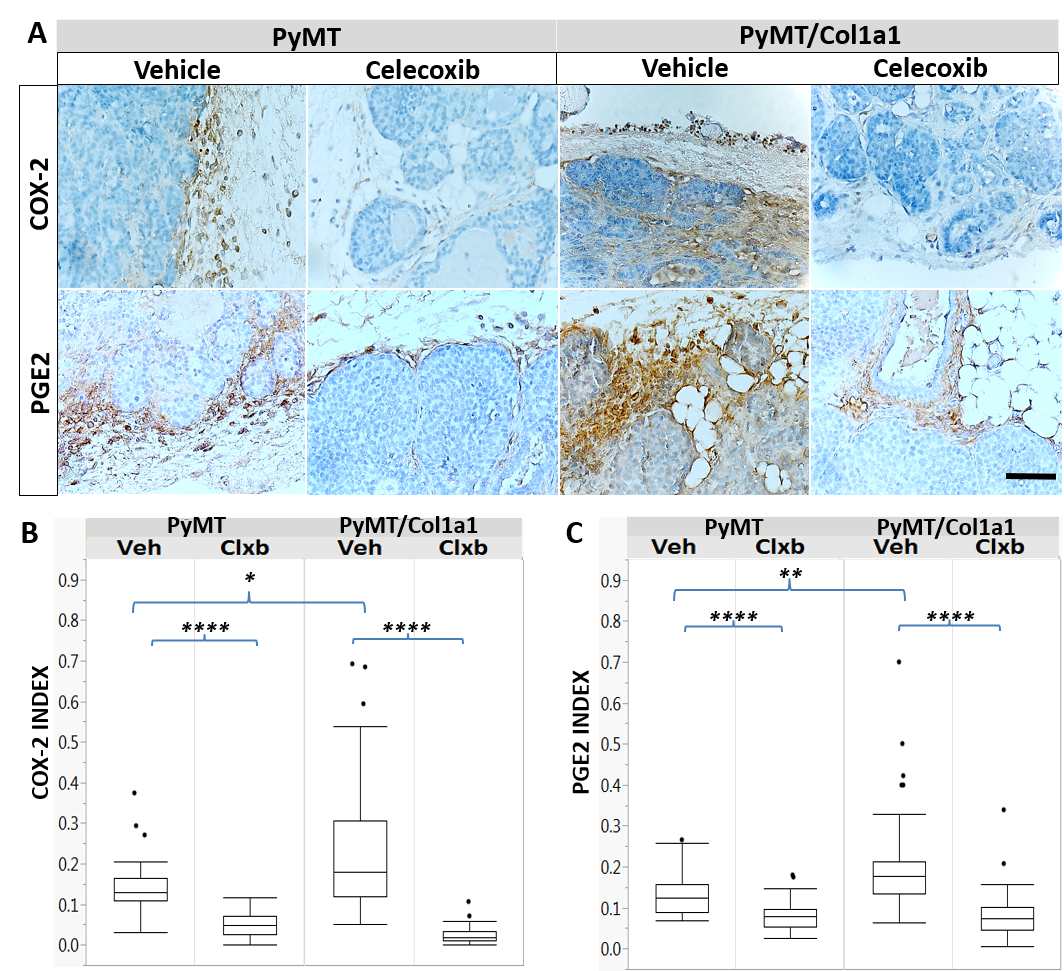

Supplement: Additional file 3: Figure S3. — Celecoxib diminishes COX2 and PGE2. a IHC images of COX-2 and PGE2 (DAB) counterstained with hematoxylin; ×40 objective; scale bar = 50 um. b, c Index was calculated by dividing amount of positive-stained cells over total amount of cells. Graphs depict raw data. b COX-2 levels are elevated in PyMT/Col1a1 tumors. Celecoxib diminishes COX-2 levels in PyMT and PyMT/Col1a1 tumors. c PGE2 levels are elevated in PyMT/Col1a1 tumors and celecoxib diminishes PGE2 levels in PyMT and PyMT/Col1a1 tumors; *p < 0.05, **p < 0.01, ****p < 0.0001; n = 5 mice per arm; at least 8 image fields analyzed per 2–3 tumors per animal; mixed linear model. (PNG 1241 kb) [file 13058_2016_695_MOESM3_ESM.png]

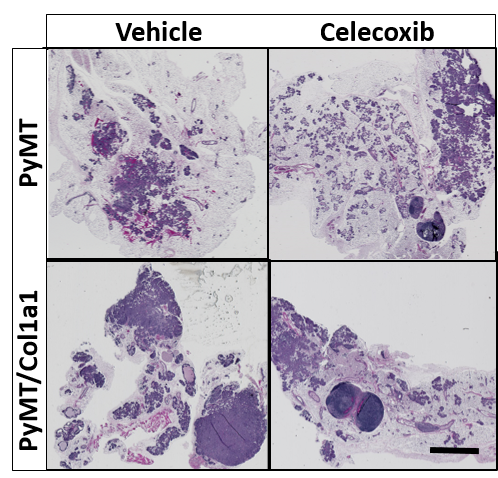

Supplement: Additional file 4: Figure S4. — Histology of mammary tumors after FDG-PET imaging in the early treatment study. Hematoxylin and eosin stained sections of mammary tumors from PyMT and PyMT/Col1a1 mice at 9 weeks of age, treated with celecoxib or vehicle. Histological images confirm the difference in tumor growth between PyMT and PyMT/Col1a1 mice and tumor growth reduction in PyMT/Col1a1 mice when treated with celecoxib prior to tumor formation; ×4 objective; scale bar = 0.5 mm. (PNG 430 kb) [file 13058_2016_695_MOESM4_ESM.png]
